# Chronic exposure to multiple stressors alters the salivary proteome of piglets

**DOI:** 10.1371/journal.pone.0286455

**Published:** 2023-05-26

**Authors:** Sara Prims, Xaveer Van Ostade, Miriam Ayuso, Martin Dom, Geert Van Raemdonck, Steven Van Cruchten, Christophe Casteleyn, Chris Van Ginneken

**Affiliations:** 1 Laboratory of Comparative Perinatal Development, Department of Veterinary Sciences, Faculty of Pharmaceutical, Biomedical and Veterinary Sciences, University of Antwerp, Antwerp, Belgium; 2 Laboratory of Protein Chemistry, Proteomics and Epigenetic Signalling (PPES), Department of Biomedical Sciences, Faculty of Pharmaceutical, Biomedical and Veterinary Sciences, University of Antwerp, Antwerp, Belgium; 3 Center for Proteomics (CfP), University of Antwerp, Antwerp, Belgium; University of Illinois, UNITED STATES

## Abstract

Monitoring chronic stress in pigs is not only essential in view of animal welfare but is also important for the farmer, given that stress influences the zootechnical performance of the pigs and increases their susceptibility to infectious diseases. To investigate the use of saliva as a non-invasive, objective chronic stress monitoring tool, twenty-four 4-day-old piglets were transferred to artificial brooders. At the age of 7 days, they were assigned to either the control or the stressed group and reared for three weeks. Piglets in the stressed group were exposed to overcrowding, absence of cage enrichment, and frequent mixing of animals between pens. Shotgun analysis using an isobaric labelling method (iTRAQ) for tandem mass spectrometry performed on saliva samples taken after three weeks of chronic stress identified 392 proteins, of which 20 proteins displayed significantly altered concentrations. From these 20 proteins, eight were selected for further validation using parallel reaction monitoring (PRM). For this validation, saliva samples that were taken one week after the start of the experiment and samples that were taken at the end of the experiment were analysed to verify the profile over time. We wanted to investigate whether the candidate biomarkers responded fast or rather slowly to the onset of chronic exposure to multiple stressors. Furthermore, this validation could indicate whether age influenced the baseline concentrations of these salivary proteins, both in healthy and stressed animals. This targeted PRM analysis confirmed that alpha-2-HS-glycoprotein was upregulated in the stressed group after one and three weeks, while odorant-binding protein, chitinase, long palate lung and nasal epithelium protein 5, lipocalin-1, and vomeromodulin-like protein were present in lower concentrations in the saliva of the stressed pigs, albeit only after three weeks. These results indicate that the porcine salivary proteome is altered by chronic exposure to multiple stressors. The affected proteins could be used as salivary biomarkers to identify welfare problems at the farm and facilitate research to optimise rearing conditions.

## Introduction

Pigs are exposed to several stressors in their lives, such as regrouping (e.g. [1, 2]), castration (e.g. [3]), and road transport (e.g. [4]). Other stressors like restricted floor space [5, 6], inappropriate light and temperature [7], or lack of sufficient and/or qualitative enrichment [8, 9] can also occur. When the stressor exceeds a certain threshold in duration and magnitude, the body’s homeostasis is disturbed. The equilibrium can be re-established by behavioural and physiological adaptive responses or by removing the stressor. However, failure to generate sufficient adaptive responses could lead to chronic stress, implying compromised animal welfare and suboptimal pig production due to a suppressed immune system (e.g. [10, 11]), reduced zootechnical and breeding performance (e.g. [12–16]). Thus, identifying and eliminating stress is essential for both the pig and the farmer.

To evaluate chronic exposure to stressors in pigs, behavioural assessments are often implemented despite being labour-intensive and difficult to interpret (e.g. [17]). Alternative methods, such as evaluating hyperkeratosis and ulcer formation in the stomach, are only feasible *post mortem* [18]. Cortisol concentrations in hair can be a good indicator of chronic stress in pigs [19, 20]. However, the sample preparation is labour-intensive and time-consuming. As a result, an objective, fast method that preferably relies on quantifiable biomarkers is sought after. Such biomarkers are routinely examined in blood, the most studied biological fluid in the past (e.g. [21, 22]). Unfortunately, blood sampling requires trained staff and, more importantly, induces stress on the pig [23]. In contrast, saliva collection does not require qualified personnel, is non-invasive, and is stress-free. Moreover, the proteome of pig saliva contains a wealth of proteins, as we demonstrated before [24], from which some could serve as biomarkers for chronic exposure to stressors. Therefore, saliva was investigated as a potential biological sample to detect stress in pigs and is nowadays preferred over blood analysis (e.g. [25–27]). Most studies, however, focused on acute short-term stress, whereas only limited experiments investigated salivary profile differences in chronically stressed pigs. Cortisol (e.g. [25]), chromogranin A [8, 28], and serum amyloid A [29] have already been targeted in porcine saliva using antibody-based techniques concerning chronic stressor exposure. At the same time, only one study investigated the salivary proteome in an untargeted way using high-resolution mass spectrometry (MS) [30]. The latter looked for candidate biomarkers in relation to compromised animal welfare due to lameness [30]. To our knowledge, no data on the salivary proteome of pigs in which chronic stress was experimentally induced are available. Therefore, the present study aimed to compare the salivary proteome of piglets chronically exposed to different stressors, including overcrowding, deprivation of cage enrichment, and frequent mixing of non-familiar individuals, with that of control piglets that were left undisturbed. We used isobaric tags for relative and absolute quantification (iTRAQ) in combination with a sensitive, high-resolution orbitrap MS/MS method. Because antibody-based assays on porcine proteins are scarce, a subset of the identified salivary proteins found in different relative concentrations was further validated using parallel reaction monitoring (PRM).

## Materials and methods

### Animals and housing

**Fig 1:**
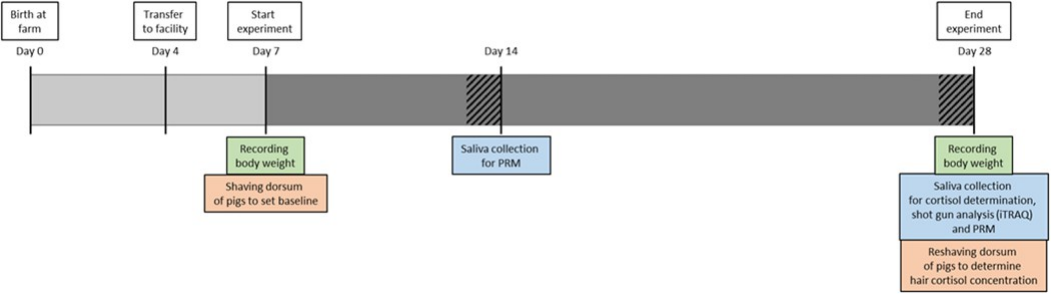
Timeline of the experiment. From the age of 7 days until the end of the experiment at the age of 28 days, animals of the stressed group were exposed to three stressors, including overcrowding, deprivation of cage enrichment, and mixing with unfamiliar animals (dark grey). The latter was paused for 24h before saliva collection (hatched area). The time points at which the body weight was recorded for weight gain (green), the hair was shaven for its cortisol determination (orange), and saliva was sampled for iTRAQ analysis, cortisol determination, and PRM validation (blue) are indicated.

All experiments were approved by the Ethical Committee for Animal Experiments of the University of Antwerp, Belgium (2016–41) and according to the European Directive (2010/63/EU). Twenty-four healthy female piglets (Belgian Landrace × Piétrain), with an average body weight within one standard deviation from the mean, born from eight sows, were transported from a local farm to the University of Antwerp at the age of 4 days (Fig 1). All animals received an intramuscular iron injection (Iron(III) Dextran, 200 mg/piglet, Uniferon, Pharmacosmos, Holbaek, Denmark) on day 3. No antibiotics or vaccines were administered prior or during the studied period. They were allowed to adjust to the new environment until the start of the experiment at the age of 7 days. Only female piglets were selected. The stressful event of castration for male piglets could interfere with our study design because it may not merely cause an acute activation of the hypothalamic-pituitary-adrenal (HPA) axis [31] but could also sensitise the pigs for later stressors [32, 33]. On the other hand, oestrogen enhances HPA function, possibly making female piglets more susceptible to stress [34]. The piglets were housed on commercial brooders (Rescue Decks^®^, S&R Resources LLC, Mason, USA) and artificially reared on milk formula (BIGGILAC PL+, AVEVE, Antwerp, Belgium), which was provided ad libitum until the age of 28 days (end of the experiment). They had free access to water and were maintained under standard environmental conditions (12h/12h light/dark cycle, temperature adjusted to age).

Litter-matched piglets were randomly assigned to either the control group (n = 8) or the stress group (n = 16). The latter group was simultaneously exposed to three known stressors: overcrowding (e.g. [5, 35]), mixing with unfamiliar piglets (e.g. [16, 36]), and absence of cage enrichment (e.g.[8]) for 21 days, from the age of 7 days until the age of 28 days. The animals of the stressed group were housed in two subgroups of eight animals, each subgroup in a smaller brooder reducing the stocking density to 0.10 m^2^/animal, bringing it under the European guideline of a minimum of 0.15 m^2^/piglet (< 10 kg; 2001/88/EC). Additionally, throughout the experiment on 32 random time points during the daytime, piglets of the stressed group were exchanged between the two brooders to disturb the social hierarchy and induce social stress. Finally, environmental enrichment was not provided to piglets in the stressed group. The control piglets were housed in groups of four at a density of 0.29 m^2^/animal with balls and ropes as environmental enrichment. Animals were observed twice a day, paying attention to behaviour, body condition, lesions, and faecal composition.

### Physiological parameters

To determine the effectiveness of the applied stressors, some previously established chronic stress indicators were used, i.e., bodyweight gain and cortisol concentrations in saliva and hair.

Bodyweight was distributed evenly between both experimental groups when transported to our facility on day 4 (control group: 1.89 ± 0.40 kg; stressed group: 2.05 ± 0.41 kg). Bodyweight was recorded at the start of the experiment at the age of 7 days and at the end when the animals were 28 days old (Fig 1). Saliva was collected at 14 days of age (early time point in the experiment) and 28 days of age (end of the experiment) between 8:30 am and 9:30 am. The piglets were not exposed to mixing stress 24 h before saliva collection to avoid acute stress. Piglets were allowed to chew on a synthetic cylindrical collection pad mounted on a handle (Micro·SAL, Oasis Diagnostics) validated for cortisol analysis [37]. Saliva was recovered from this pad by placing it in a syringe-like compression chamber, pushing the plunger firmly downwards, and transferring the saliva into a clean Eppendorf tube. All samples were aliquoted and stored at -80°C until further analysis.

Cortisol concentrations were determined in duplicate in a single assay using a commercially available cortisol saliva ELISA (IBL-International, Hamburg, Germany) validated for pig saliva, following the manufacturer’s guidelines [38].

To determine cortisol accumulation in the hair during the three-week experiment, the dorsum (about 35 cm x 10 cm) of each piglet was shaved at the start of the experiment (7 days old) with clippers, and the hair was discarded to set the baseline. The dorsum was chosen since mechanical forces resulting in scratching and rubbing that could elevate cortisol concentrations in hair locally infrequently occur in this region [39]. After three weeks (28 days old), at the end of the experiment, the dorsal area was shaved again. The collected hairs, in which cortisol had accumulated during the experiment, were washed twice for 3 min with 10 mL isopropanol on an orbital shaker to remove dust and sebum [40]. After 5 days of drying, the hair samples were ground using a pestle and mortar. Because cortisol levels are lower in hair from the craniodorsal area than in hair from the dorsolumbar region, the whole sample was homogenised before further analysis [19]. From this sample, 50 mg was taken and added to 1.8 mL methanol. After incubation for 24 h, the samples were centrifuged for 15 min at 1500 g [40]. From the supernatant, 1.3 mL was lyophilised and resuspended in 300 μL of phosphate-buffered saline. Cortisol concentrations were determined using the same ELISA as used for the determination of cortisol levels in saliva. Intra-assay coefficients of variation for both cortisol assays were < 5%.

### Shotgun proteomics on saliva

#### Sample preparation

Proteins in the individual saliva samples were labelled using iTRAQ-labels, reagents, and buffers. Working with the 8-plex kit allowed us to pool eight samples and simultaneously analyse them in one run since the mass spectrometer is able to distinguish proteins from different samples after tandem MS analysis [41]. Consequently, three parallel analyses were performed so the relative abundance of all 24 individually labelled samples could be determined. Each parallel analysis contained samples of both the control and the stressed group. The protocol was according to the manufacturer’s guidelines (Applied Biosystems Sciex Inc., MA, USA) and similar to what we described previously [24]. In brief, the total protein concentration of all saliva samples was determined using a bicinchoninic acid assay (BCA, Thermo Scientific, San Jose, CA, USA). Volumes containing 100 μg of protein were purified by means of acetone precipitation to discard any salts and lipids. The resulting protein pellets were resuspended in 500 mM triethylammonium bicarbonate (TEAB). Hydrogen bonds were disrupted, and disulphide bonds were reduced using 2% sodium dodecyl sulfate (SDS) and 50 mM tris-(2-carboxyethyl) phosphine (TCEP), respectively. The samples were incubated with 200 mM methyl methanethiosulfonate (MMTS) to alkylate thiols reversibly. Subsequently, trypsin (Promega, Leiden, The Netherlands) was added in a one-to-ten ratio (g/g) to digest proteins during the overnight incubation step at 37°C. Afterward, the eight samples were labelled using the eight different iTRAQ reagents. All eight individually labelled samples were combined, resulting in one batch for further analysis by two-dimensional liquid chromatography combined with tandem mass spectrometry (2D-LC-MS/MS).

#### First-dimensional separation

The combined sample was fractionated in a first dimension by strong cation exchange (SCX) chromatography using a Waters Alliance e2695 HPLC system with Photo Diode Array Detector (Waters Corporation, Zellik, Belgium). After acidification to a pH of 2.7, the sample was loaded onto a polysulfoethyl-aspartamide SCX-column (2.1 mm x 200 mm; 5 μm particles; PolyLC Inc., Columbia, MD, USA). Three different solvents (solvent A: 10 mM KH_2_PO_4_, 20% acetonitrile (ACN) (pH 2.7); solvent B: 10 mM KH_2_PO_4_, 650 mM KCl, 20% ACN (pH 2.7) and solvent D: 10 mM KH_2_PO_4_, 650 mM KCl, 20% ACN (pH 4.7)) were used to separate the combined peptide sample according to their charge. First, only solvent A was used for 10 min followed by a salt gradient (7.5–30%) of solvent B for 45 min and a pH gradient (30–100%) of solvent D for 15 min, with a final 5 min step of only solvent D to eluate highly charged peptides. During the entire gradient, a flow rate of 200 μL/min was kept constant. In total, ten fractions were collected, of which the total peptide concentration was determined using the area under the curve (AUC). These were lyophilised and frozen until further analysis.

#### Second-dimensional separation and Q-exactive orbitrap MS/MS analysis

The ten SCX fractions were resuspended in LC-MS H_2_O to desalt the peptides using solid phase extraction (SPE). GracePure^™^ SPE C18 Columns (W. R. Grace & Co.-Conn., Maryland, USA) were placed onto a vacuum manifold and subsequently conditioned (three times with 100 μL methanol) and equilibrated (twice with 100 μL LC-MS H_2_O) before the fractions were loaded (two times, reloading the eluate), washed (twice with 100 μL (20% methanol, 80% LC-MS H_2_O)) and eluted (twice with 100 μL (40% methanol, 40% ACN, 20% 0.1% HCl in LC-MS H_2_O)). The eluted peptides were subsequently lyophilised and frozen until further analysis. Each SCX fraction was separated in a second dimension by RP-C18 chromatography on an Easy nanoLC system using an Acclaim C18 PepMap^®^100 column (75 μm x 2 cm, 3 μm particle size) connected to an Acclaim PepMap^®^ RSLC C18 analytical column (50 μm x 15 cm, 2 μm particle size) (Thermo Scientific). Before loading, the vacuum-dried peptide pellets were dissolved in mobile phase A (2% ACN and 0.1% formic acid (FA)). Of each SCX fraction, 1 μg of peptides were loaded onto the column. One technical replicate was performed for each sample. A linear gradient of mobile phase B (0.1% FA in 95% ACN) from 2% to 45% in 55 min, followed by a steep increase to 100% mobile phase B in 5 min, was used at a flow rate of 300 nL/min. Liquid chromatography was followed by MS, performed on a Q-Exactive Plus mass spectrometer equipped with a nanospray ion source (Thermo Scientific). The high-resolution mass spectrometer was set up in an MS/MS mode in which a full scan spectrum (350 to 1850 m/z, resolution 70,000) was followed by a maximum of five high energy collision activated dissociation (HCD) tandem mass spectra (100 to 2000 m/z). The normalised collision energy was set at 33%. A dynamic exclusion list of 15 s for data-dependent acquisition was applied. The mass spectrometry proteomics data have been deposited to the ProteomeXchange Consortium via the PRIDE [42] partner repository with the dataset identifier PXD037193 (https://www.ebi.ac.uk/pride/archive).

#### Database searching

All generated MS/MS spectra were analysed by means of MaxQuant software version 1.6.1 [43] using the *Sus scrofa* database that was generated based on both the characterised porcine proteins and the porcine genome (*Sus scrofa* database (reviewed + unreviewed), generated from UniProt (2020/03/30), 120,806 entries). Analysis was performed based on trypsin digestions. Methyl methanethiosulfonate binding to cysteine and iTRAQ 8-plex labelling of lysine and the N-terminus were specified as fixed modifications. Oxidation of methionine and iTRAQ 8-plex labelling of tyrosine were set as variable modifications. Only proteins or protein groups with at least two unique peptides were further investigated. MaxQuant sometimes reports protein groups instead of a single protein. These groups are clusters of proteins that could not be distinguished from each other based on the identified peptides [43]. The leading protein of this group is the protein with the best match and the protein that is referred to in this manuscript. The false discovery rate (FDR) was set at 1% and guarded using a reversed decoy database. All human keratins and other possible contaminants were removed from the output list. A BLAST analysis was performed on all uncharacterised proteins (BLASTP 2.8.0+, All non-redundant GenBank CDS translations+PDB+SwissProt+PIR+PRF excluding environmental samples from WGS projects Program, *Sus scrofa* (taxid:9823)) [44]. These proteins are further identifiable by the word BLAST in front of their names. The fold-change difference of each protein was calculated, proteins whose relative abundance had a fold-change of at least 1.5 where further investigated.

#### Statistical analysis

Mixed models were fitted to identify differences between experimental groups for the following parameters: body weight gain, cortisol concentrations in saliva and hair, and relative concentrations of specifically identified proteins. The sow was implemented as a random factor to account for the dependence of littermates. To determine differences between concentrations of specific salivary proteins, the different iTRAQ runs were added as a random factor for normalisation since three parallel runs were necessary to analyse all 24 samples. To meet normality and homoscedasticity assumptions, data from hair and salivary cortisol levels and some specific salivary protein concentrations were log-transformed. A Spearman rank correlation test was performed to investigate the relationship between all assessed parameters. All data were analysed using JMP^®^ Pro 13 (SAS Institute Inc., North Carolina, USA) and reported as medians and 25^th^/75^th^ percentiles. A *P*-value ≤ 0.05 was considered statistically significant.

### Candidate biomarker validation using PRM

To confirm the salivary protein profile differences between stressed and control piglets, eight of the proteins from the discovery iTRAQ-experiment were selected for further validation. Since no commercially available ELISAs or antibody-based quantification techniques were available for the selected porcine proteins, we opted to validate these eight selected proteins using parallel reaction monitoring (PRM). This is a targeted mass spectrometry approach, often used to validate candidate biomarkers. During this analysis specific peptides are selected and fragmented. The abundance of each fragment is detected with very high sensitivity and specificity (e.g. [45]).

#### Selection of proteins

The selection of proteins was based on different parameters. Preferably the proteins had a high fold-change difference and/or a small *P*-value and/or had a known function or involvement in processes that are affected by stress, such as immunity, feeding behaviour, or reproduction capacity (e.g. [10–15]). Moreover, some proteins were chosen because they had previously been reported to show altered concentrations in saliva after acute stress or compromised welfare conditions in pigs or other species [30, 46–48]. The final criterium was the possibility of detecting an adequate amount of proteotypic peptides for the target protein group during PRM optimisation, i.e., shotgun analysis and unscheduled PRM. For each of the final eight target proteins, three to five of these proteotypic peptides were listed to be monitored using an inclusion list (S1 Table). These are the peptides that will be selected during the scheduled PRM analysis based on their known retention time and m/z for relative quantification.

Peptides from serotransferrin were also relatively quantified to detect potential blood contamination (e.g. [49]). Chewing and oral problems can cause small wounds in the piglet’s oral cavity through which blood can leak during saliva collection. Some candidate biomarkers, like alpha-2-HS-glycoprotein, but also cortisol, are present in low concentrations in saliva but in much higher concentrations in blood (e.g. [50, 51]). Therefore, even small amounts of blood contamination can result in artificially high levels of these components in saliva [52]. Serotransferrin is also a protein that is present in higher concentrations in the blood than in saliva. Consequently, we used it as a marker for blood contamination. Although it is known that several factors, such as age, gonadal hormones, salivary flow rate and chewing also affect serotransferrin levels in saliva [53], it is suggested as the best indicator for blood contamination [49]. Noteworthy is that all saliva samples were visually inspected, and no discoloured (pink or red) samples were included in the analysis.

Finally, specific peptides for two additional control proteins were added to the inclusion list. These proteins are apomucin and sulfhydryl oxidase, which are chosen to identify large differences in the background proteome due to sampling, individual sample preparation, or parallel analysis. Candidates for salivary control proteins are amylase, mucins, albumin, or IgA. However, the abundance of all of these proteins except for mucins is altered by acute stress [47, 54, 55]. Therefore, we opted for apomucin, which was previously detected in porcine saliva by our research group [24]. The variation in the abundance of this protein between animals was small. However, since it was more abundant in submandibular and sublingual secretions than in parotid saliva, chewing could introduce more variation of the concentration in whole saliva present in the oral cavity. The reason for this is that chewing increases the contribution of the parotid gland to the whole saliva therefore diluting and decreasing the concentration of apomucin in whole saliva [56]. Thus, we included sulfhydryl oxidase as a second protein with low variation between animals and with equal concentrations in submandibular/sublingual and parotid saliva [24].

To see whether there was a difference in the abundance profile over time in these eight candidate biomarkers, we determined their relative abundance in salivary samples taken from the piglets at the age of 14 days (one week after the start of the experiment) and 28 days (three weeks after the start).

#### Sample preparation

All saliva samples of days 14 and 28 were enzymatically digested according to the following protocol. All samples where thawed and volumes containing 50 μg of protein were denatured at 90°C for 5 minutes. The samples were allowed to cool down and 2.5 μL of 50 mM of TCEP was added followed by a 1-hour incubation step at 55°C. To alkylate thiols irreversibly, 5 μL of 375 mM iodoacetamide (Biolsolve BV, Valkenswaard, The Netherlands) were added to each sample and incubated for 30 minutes. All protein mixtures were purified by acetone precipitation. The obtained protein pellets were resuspended in 500 mM TEAB. Subsequently, trypsin was added to digest proteins during an overnight incubation at 37°C. All peptide samples were lyophilised and frozen until further purification using C18 spin columns (Thermo Scientific), according to the manufacturer’s instructions, except using FA (Merck KGaA, Darmstadt, Germany) instead of trifluoroacetic acid. The purified digest was lyophilised again and frozen.

#### Nano reversed phase liquid chromatography and mass spectrometry

The digested peptides were reconstituted in 0.1% FA and analysed on a Q-Exactive Plus mass spectrometer (Thermo Scientific) connected to a nanoAcquity UPLC system (Waters Corporation). For each sample, a tryptic digest of peptides equivalent to 0.5 μg total protein was loaded on a 200 cm micro Pillar Array Column (μPAC^™^, PharmaFluidics, Ghent, Belgium) retrofitted to a NanoSpray Flex source. Peptides were eluted at a flow rate of 750 nL/min using the following gradient: 1% to 40% ACN in 0.1% FA/H_2_O for 30 min, 40% to 99% ACN for 5 min, 99% to 1% ACN for 5 min and 35 min at 1% ACN in 0.1% FA/H_2_O. Analytes were transferred to the gaseous phase with positive ion electrospray ionisation at 1.9 kV. Precursors were targeted with a 0.8 m/z isolation window around the m/z of interest. Precursors were fragmented in high-energy collisional dissociation (HCD) mode with normalised collision energy of 28. A single MS1 scan was performed at a mass resolution of 17,500, an automatic gain control (AGC) target of 10^6^ ions and a maximum C-trap fill time of 200 ms. Subsequently, 10 PRM scans were performed at a resolution of 70,000, an AGC target of 10^5^ ions and a maximum injection time of 250 ms. Retention-time scheduling of PRM (sPRM) was adopted, which allowed for the analysis of all peptides in a single LC-MS analysis.

#### Data analysis

Skyline 20.1 [57] was used to analyse all PRM raw data. Only peptides with a idotp score ≥ 0.8 were included, meaning that they were proven to be of good quality after comparison of the experimental transitions to the theoretical spectral library that was generated by Prosit [58]. All transitions for one peptide were added up. The sums of the proteotypic peptides of one protein were averaged to indicate its abundance within each sample. Mixed models were fitted using JMP^®^ Pro 13 to identify differences between the stressed and control group on the one hand and the two sampling time points on the other hand. The interaction term between groups and time points was added as a fixed factor. The sow was included as a random factor to account for the dependence of littermates. The same piglets were sampled on days 14 and 28, so the piglet was nested in the sow and added as a random factor. This initial model was simplified by removing all non-significant effects using stepwise backward modelling. To meet normality and/or homoscedasticity assumptions, all data were log-transformed. Correlations were investigated using the non-parametric Spearman’s assay. Correlations with a Spearman’s rank correlation coefficient (ρ) higher than 0.4 (0.6 or 0.8) or smaller than -0.4 (-0.6 or -0.8) were moderate (strong or very strong) correlations. A *P*-value ≤ 0.05 was considered statistically significant.

## Results

### Physiological parameters

The piglets that were exposed to the stressors gained significantly less weight (*P* = 0.021) during the three-week experiment compared to the control piglets (Table 1). Cortisol concentrations in saliva at day 28 were not significantly different (*P* = 0.447) between both groups. The stressed group had significantly higher concentrations of cortisol (*P* = 0.005) in their hair compared to their control littermates. The total concentration of proteins in saliva did not differ between both groups and/or between ages (*P* = 0.531). Weight gain and cortisol concentrations in hair showed a significant negative correlation (*P* = 0.036, *ρ* = - 0.430). At the same time, neither of these parameters correlated significantly with cortisol levels in saliva at day 28 (*P* = 0.904, *ρ* = 0.026 and *P* = 0.929, *ρ* = 0.019, respectively).

**Table 1:** Physiological parameters.

	Weight gain (kg)	Concentration cortisol (μg/dL saliva)	Concentration cortisol (pg/mg hair)	Protein concentration in saliva (day 14) (μg/mL)	Protein concentration in saliva (day 28) (μg/mL)
**Control group**	7.58 (6.79–8.22)*	0.25 (0.21–0.39)	75.60 (69.95–78.42)*	10352 (8337–11485)	6915 (5432–8305)
**Stressed group**	6.43 (5.94–7.20)*	0.30 (0.27–0.51)	87.29 (78.55–99.61)*	9040 (8142–10658)	7812 (5783–16690)

Weight gain over 21 days, cortisol concentrations in saliva at day 28, cortisol accumulation in the hair over 21 days (measured on day 28), and total protein concentration in saliva at day 14 and day 28. Values are displayed as the median of the group, with the 25^th^ and 75^th^ percentiles shown between brackets. Values that differed significantly between the control and the stressed group are indicated with an asterisk (*P*-value < 0.05).

### Identified proteins using iTRAQ

In total, 421 protein groups were identified based on at least two unique peptide identifications. After the removal of all human keratins and possible contaminants, 392 proteins remained, of which 13 were uncharacterised proteins (S2 Table). Of this protein list, 255 proteins were detectable in all three parallel iTRAQ runs, including samples of both the control and stressed groups. In total, the abundance of 26 proteins was up-or downregulated with a fold change of ≥ 1.5 between the saliva of control and stressed animals. Further statistical analysis of these 26 proteins using mixed models confirmed that 20 proteins showed a significant difference in salivary concentration between both treatment groups. Six of these 20 proteins were found in lower concentrations in the saliva of stressed animals, while 14 proteins were upregulated (Table 2).

**Table 2:** Identified salivary proteins with a significant fold difference.

	Protein name	UniProt ID of lead protein	Number of unique peptides detected	Mol. weight [kDa]	Fold change difference	Fold change (stressed/ control)	Mixed model (*P* -value)
1	Odorant-binding protein*	P81245	2	17.71	2.22	Down	0.002
2	Long palate lung and nasal epithelium protein 5*	A7J153	10	54.11	2.18	Down	0.001
3	Chitinase*	I3LL32	9	51.97	1.6	Down	0.003
4	BLAST: Vomeromodulin-like protein*	F1S501	10	49.60	1.57	Down	0.001
5	Lipocalin-1*	P53715	9	19.37	1.56	Down	0.010
6	Vitelline membrane outer layer protein 1 homolog	F1RFV3	3	21.53	1.53	Down	0.010
7	Haemoglobin subunit beta	F1RII7	6	16.17	2.07	Up	0.008
8	Haemoglobin subunit alpha	P01965	9	15.04	1.95	Up	0.026
9	BLAST: Basic salivary proline-rich protein 1	A0A4X1U5H6	4	25.88	1.86	Up	0.038
10	CD5 molecule like	F1RN76	2	59.28	1.84	Up	0.001
11	Biliverdin reductase B	I3LQH7	4	22.21	1.72	Up	0.001
12	Basic proline-rich protein	Q95JC9	2	46.02	1.7	Up	0.009
13	Heat shock protein family A (Hsp70) member 9	F1RGJ3	2	70.12	1.68	Up	0.014
14	Parotid secretory protein*	Q6XZB6	9	25.98	1.66	Up	0.012
15	Apolipoprotein A-II	Q7YRR7	3	11.11	1.61	Up	0.001
16	Albumin	F1RUN2	25	67.14	1.6	Up	< 0.001
17	BLAST: Basic proline-rich protein 1	A0A5G2R9V5	3	17.54	1.59	Up	0.007
18	Carbonic anhydrase*	B7X727	9	36.31	1.57	Up	0.020
19	Alpha-2-HS-glycoprotein*	F1SFI7	5	38.79	1.55	Up	< 0.001
20	Ig lambda chain C region	P01846	3	11.00	1.53	Up	< 0.001

List of proteins of which a significant fold change difference was seen during the exploration phase using iTRAQ labels. If the fold change difference is described as up, this indicates that the values were higher in the saliva of stressed animals compared to those of the control group. A BLAST analysis was performed on all uncharacterised proteins, which are identifiable by the word BLAST in front of their names. Asterisks indicate the proteins that were further validated using PRM.

A functional analysis for the 392 filtered proteins was performed with the gene ontology database Panther (http://www.pantherdb.org/; Version 17.0). First, the genes that encoded the listed proteins were sought. From the 392 protein entries, 280 could be matched with their encoding gene. These genes could be assigned to 10 different molecular functions. Most of the genes encoded for proteins that had binding (40.1%) or catalytic functions (35.6%). These 280 recognisable genes were involved in 16 different biological processes. Of these, more than 80% were involved in cellular (50.1%) or metabolic (33.1%) processes, while only a small fraction of these genes encoded proteins that were involved in immunity (5.9%), growth (2.2%) or reproductive processes (0.6%).

### Biomarker validation using PRM

**Fig 2:**
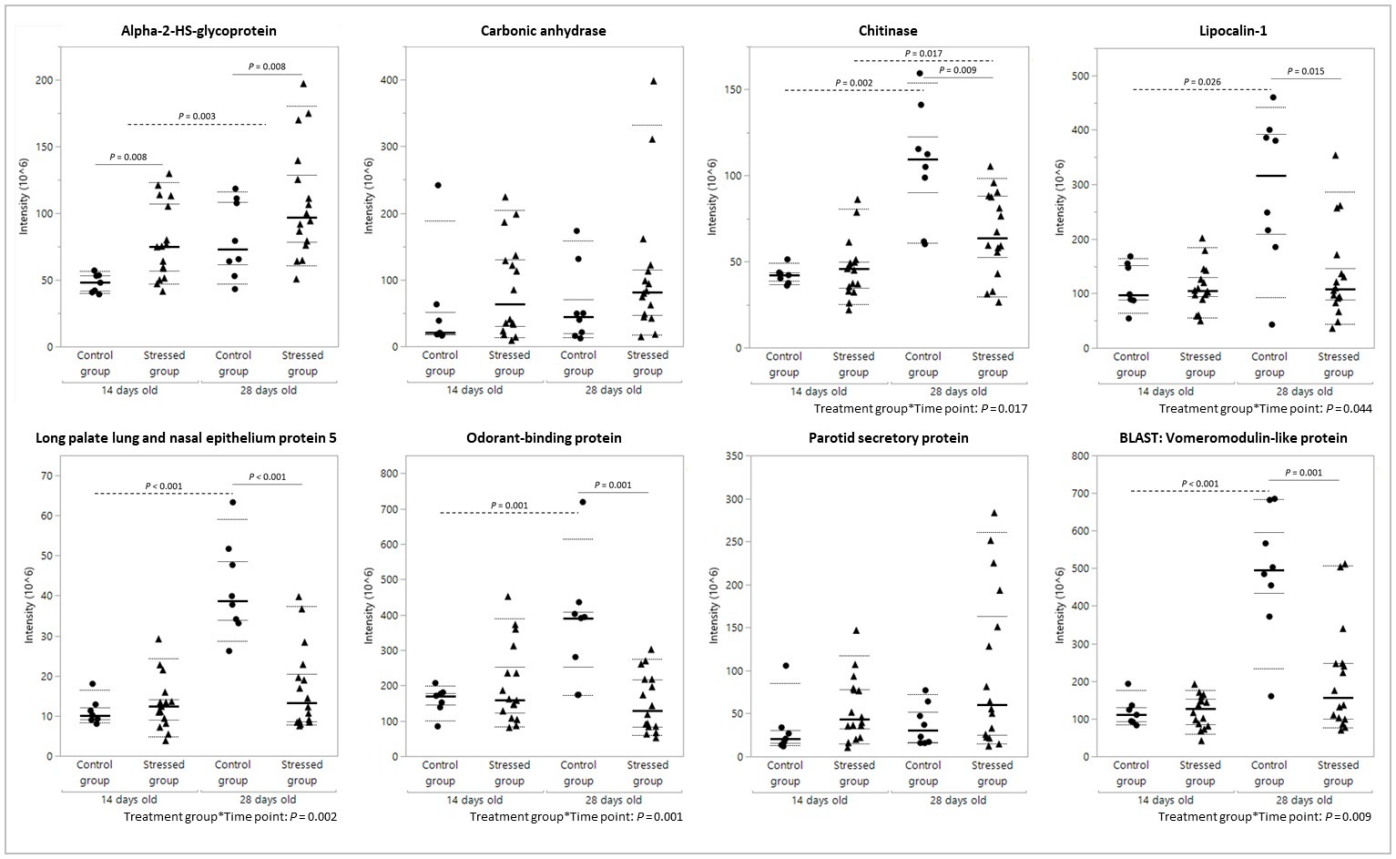
The abundance of proteins validated by PRM. Statistically significant differences between samples taken at the age of 14 days and samples from day 28 are highlighted by dashed lines. Significant differences between the control group (circles) and the stressed group (triangles) are indicated with a full line. Significant interaction terms are placed underneath each graph. For each group (control group (*n* = 8); stressed group (*n* = 16)), the median (thick line), the 25^th^ and 75^th^ percentiles (thin lines), and the 5^th^ and 95^th^ percentiles (dotted lines) are shown.

Eight proteins from the list of 20 were selected for further analysis using PRM. Alpha-2-HS-glycoprotein is a protein that was found in higher concentrations in the saliva of 4-week-old stressed animals after shotgun investigation. This observation was confirmed by the targeted validation (*P* = 0.003) (Fig 2). This holds true for samples taken one week after the start of the experiment as well as at the end. It is noteworthy that the concentration of this protein rose with age (*P* = 0.008) in both the stressed and the control groups.

In contrast, the differences in concentration of the two other selected proteins with an upregulation in the saliva of stressed animals were not confirmed by PRM. Carbonic anhydrase and the parotid secretory protein did not show any difference between both experimental groups (*P* = 0.265 and *P* = 0.129, respectively) or between the different sampling points (*P* = 0.363 and *P* = 0.246, respectively).

The five other proteins that were validated using PRM all had a significant interaction of experimental groups and time points, meaning that the effect of stress was not the same at both time points. Post hoc analysis of the concentrations of chitinase showed a rise in concentration from the age of 14 days to 28 days (*P* = 0.002) in the saliva of the control group. This rise was less pronounced in the stressed group (*P* = 0.017), resulting in a significant difference in chitinase concentration between the two experimental groups on day 28 (*P* = 0.009) but not on day 14. Lipocalin-1, long palate lung and nasal epithelium protein 5, odorant-binding protein, and BLAST: vomeromodulin-like protein all had a similar profile. The latter was an uncharacterised protein that, after a BLAST analysis, appeared to be a homolog of the vomeromodulin-like protein (*Bison bison*). All four proteins showed a different effect of time on both experimental groups. Only a rise in concentration over time was observed in the control group (lipocalin-1: *P* = 0.026; long palate lung and nasal epithelium protein 5: *P* < 0.001; odorant-binding protein: *P* = 0.001 and BLAST: vomeromodulin-like protein: *P* < 0.001). In the stressed group, the concentration of these proteins remained the same. Therefore, the values of these four proteins were significantly lower in the saliva of the stressed piglets compared to those of the control group, albeit only on day 28 (lipocalin-1: *P* = 0.015; long palate lung and nasal epithelium protein 5: *P* < 0.001; odorant-binding protein: *P* = 0.001 and BLAST: vomeromodulin-like protein: *P* = 0.001).

Odorant-binding protein was the only salivary candidate biomarker with concentrations that correlated to the other determined physiological parameters (S1 Fig). The concentrations of this protein on day 28 determined by PRM correlated significantly with weight gain during the experiment (*P* = 0.048, ρ = 0.408). Nevertheless, the five proteins that had a downregulation on day 28 all correlated significantly with each other. The strongest correlation was found between long palate lung and nasal protein 5 and BLAST: vomeromodulin-like protein with a Spearman’s ρ of 0.944 (*P* < 0.001). Odorant-binding protein was the only protein with a significant downregulation that correlated with proteins that showed an upregulation after iTRAQ-analysis. Although PRM analysis could not confirm the difference between both treatment groups for carbonic anhydrase and parotid secretory protein, their values did correlate negatively with those of odorant-binding protein. Alpha-2-HS-glycoprotein was the only protein that did not correlate to any of the other candidate biomarkers. However, this protein did correlate positively with the abundance of serotransferrin on day 28 (*P* < 0.001, ρ = 0.747), while this was not the case for all other proteins. It is noteworthy that the values of serotransferrin also correlated with the concentration of cortisol that was detected in these salivary samples. No significant differences were found in the abundances of the selected control proteins apomucin (age, *P* = 0.375; condition, *P* = 0.058) and sulfhydryl oxidase (age, *P* = 0.286; condition, *P* = 0.107). However, the values of serotransferrin did rise with age (*P* = 0.001). No significant differences were observed between the total protein concentrations in the saliva samples, neither between treatment groups (*P* = 0.116) nor time points (*P* = 0.531). One animal stood out since the value of sulfhydryl oxidase in its saliva was 5 times higher than the median value of the 14-day old stressed group. The same animal also had a much higher value of serotransferrin on day 14. Since none of the eight validated biomarkers displayed values that deviated this much, this observation was not considered a problem for further data analysis and interpretation. For apomucin, one animal had exceeded the range for outliers of the mean ± 2.5 times the SD. This animal of the stressed group had 3 times higher values at day 28 compared to the average detected in this group. This animal had no extreme deviating values for the other examined proteins. Only two other values could be considered outliers. These were the highest value in the stressed group on day 28 for carbonic anhydrase and lipocalin-1. However, these were not from the same animal. Follow-up studies with larger sample sizes need to verify the working ranges for the biomarkers.

## Discussion

During this experiment, the control group gained, on average, significantly more weight during the 21-day study period than the piglets in the stressed group. This is not a surprise since it is known that the average daily weight gain of pigs is reduced by stress (e.g. [12]). A reduced feed intake can cause this reduction in weight gain since different stressors are known to result in lethargy and, therefore, lower feed intake [12]. In addition, the stress system can also interact with the appetite-satiety centres of the central nervous system [59, 60]. Weight gain correlated negatively with hair cortisol concentrations. Hair from stressed pigs contained significantly higher concentrations of cortisol. In contrast, no correlation between the physiological parameters, weight gain and hair cortisol concentrations, and cortisol concentrations in saliva was found. Salivary cortisol concentrations were not significantly higher after 21 days of exposure to multiple stressors. This lack of a difference was probably due to two higher saliva cortisol values in the control group. Most likely these reflected an acute response to a stressful stimulus, rather than chronic stress, although alternative biomarkers for acute stress, such as chromogranin A or IgA (e.g. [27]) were not analysed to confirm this hypothesis. Although, chromogranin A was previously also described as a marker for chronic stress in pigs making this marker less ideal for this purpose. Nevertheless, it could have been interesting to correlate the determined parameters and the determined profile of salivary proteins to the abundance of chromogranin A. Of note, the correct timing for salivary sampling could be a point of discussion. It is possible that differences between groups might be larger if sampling occurs when baseline cortisol concentrations are low, i.e., in the evening. However, the circadian rhythm of cortisol does not mature until 20 weeks of age [26], rendering the identification of daily baseline cortisol concentration in young animals unpredictable. Additionally, a previous study demonstrated that decreased welfare leads to a blunted circadian rhythm [61], making the determination of the optimal time point for sampling challenging. However, finding salivary biomarkers that are less sensitive to acute stressors and that are not subjected to a circadian rhythm would be ideal.

In this experiment, iTRAQ-analysis identified 392 proteins in porcine saliva of which many were original identifications. Together with the proteins that our group has identified before in gland-specific saliva and those identified by other researchers, the list of identified proteins of the porcine salivary proteome is approaching 500 [24, 30, 46, 62–70]. Even though our knowledge of pig saliva is growing, this number is merely a fraction of the more than 3000 identifications of the human salivary proteome (e.g. [71–73]). Of the 392 protein identifications, the abundance of 20 proteins was different after a three-week exposure to different stressors, including overcrowding, deprivation of cage enrichment and frequent mixing of individuals between pens.

### Upregulated proteins

Fourteen proteins were found to be present in higher concentrations in the saliva of stressed animals. These include alpha-2-HS-glycoprotein, apolipoprotein A-II, basic proline-rich protein, biliverdin reductase B, carbonic anhydrase, CD5 molecule-like protein, haemoglobin subunit alpha and beta, heat shock protein family A (Hsp70) member 9, Ig lambda chain C region, parotid secretory protein, albumin and two uncharacterised proteins that had homology with basic salivary proline-rich protein 1 (*Homo sapiens*) and basic proline-rich protein 1 (*Homo sapiens*).

#### PRM-validated upregulated proteins

Alpha-2-HS-glycoprotein, also called fetuin-A, is mainly synthesized by the liver and secreted into the bloodstream. It is involved in many different pathways. It is an inhibitor of insulin receptor tyrosine kinase, has adipogenic properties, and regulates bone remodelling and calcium metabolism in bones and teeth (previously reviewed [74]). Additionally, this glycoprotein plays an acute phase protein role exhibiting an anti-inflammatory function by inhibiting the production of proinflammatory mediators in macrophages [75, 76]. Because of its versatile function, alpha-2-HS-glycoprotein has been suggested as a biomarker for several human conditions, as previously reviewed [74]. High circulating levels of alpha-2-HS-glycoprotein were also found in the serum of humans with depressive episodes and anxiety within the context of insulin resistance [77–79]. In contrast, lower concentrations were found in the bronchoalveolar lavage fluid of calves exposed to road transport and weaning [48]. Because this protein is so versatile, its concentration can be influenced by many different processes and therefore seems less specific. Another disadvantage is that the concentration of this glycoprotein could be influenced by blood contamination. Consequently, the results should be interpreted with caution. On the other hand, the values of alpha-2-HS-glycoprotein in saliva were already significantly higher after one week of exposure to the stressors. This is the only protein in this experiment that responded that fast. For this reason, this protein could be valuable as a candidate biomarker that responds relatively quickly to chronic stressor exposure. However, it should not be used as a single biomarker but rather as part of a set due to its low specificity.

The parotid secretory protein, like the basic proline-rich proteins, is a protein that is also stored in acinar granules [80] and predominantly secreted by the parotid gland [24]. The function of the parotid secretory protein is still unknown. It most probably belongs to the palate lung and nasal epithelium clone (PLUNC) family of mucosal secretory proteins that are predicted to be structurally similar to lipid-binding and host defence proteins. However, different members of this family may have different biological functions [81]. While higher saliva concentrations of these proteins have only been linked to autism spectrum disorder [82], increased secretion rates under stressful conditions could possibly be explained by beta-adrenergic stimulation [83, 84]. Although six stressed animals had much higher values of parotid secretory protein after three weeks compared to the control group, this effect was not consistent in all pigs.

The last protein for which the iTRAQ analysis indicated a positive fold-change difference is carbonic anhydrase. Carbonic anhydrase isoenzyme VI is the only secretory isoenzyme of its family that is expressed in the serous acinar cells of the parotid and submandibular glands [85]. Higher concentrations of this protein have been found in pooled saliva samples of pigs after snaring [46] and in pigs with non-infectious growth-rate retardation [70]. In contrast, others did not detect a significant effect on the carbonic anhydrase VI concentration in saliva after snare restraint. Still, they did observe an increase due to lameness [63]. This discrepancy could be attributed to the presence of two different forms of carbonic anhydrase VI in porcine saliva. In the saliva of pigs with retarded growth two forms were identified of which only the larger form of this glycoprotein (36 kDa) was present in higher concentrations. In comparison, the smaller (33 kDa), assumed partially deglycosylated form, was nearly absent [70]. In our study, PRM validation failed to confirm any increase in concentration in stressed animals since only two animals of the stressed group presented elevated concentrations of this protein. Further studies should be conducted to clarify the role of carbonic anhydrase VI as a biomarker for animal welfare.

#### Non-validated upregulated proteins

The basic proline-rich proteins upregulated in the iTRAQ analysis are predominantly secreted by the parotid gland [24]. These secretory proteins are stored in acinar granules [86]. Basic proline-rich proteins are often further cleaved into smaller fragments after secretion. This group of proteins and peptides has a role in the protection and repair of dental enamel, has antimicrobial capacities, and can bind feed components such as tannins (e.g. [87–89]) but have not been associated with stress before in pigs.

The amount of CD5 molecule-like protein in the saliva of the stressed animals was nearly twice as high as that of the control animals. This observation is in line with a previous study in which higher concentrations of this CD5 molecule-like protein were found in the saliva of lame pigs [30]. Unfortunately, this protein could not be further validated in our study since not enough specific peptides were identifiable during PRM analysis. Like the upregulation of CD5 molecule-like protein, also higher levels of haemoglobin subunit alpha and beta were detected in the saliva of lame animals [30], which is consistent with our results. These haemoglobin subunits were also found in higher concentrations in the saliva of pigs after exposure to an acute stressor, i.e. snaring restraint [46]. Snaring also led to higher concentrations of albumin in saliva, as did short road transport and 24 h isolation in a metabolic cage [47]. These findings are like the 1.6-fold upregulation of albumin observed in our study. The higher levels of albumin in stressed pigs can be the result of higher cortisol levels since it has been reported that increased concentrations of cortisol could elevate albumin production [90]. Salivary albumin concentrations were also found in higher concentrations in pigs suffering from infection and/or inflammation [62, 69]. The four proteins described above, namely CD5 molecule-like protein, haemoglobin subunit alpha and beta, and albumin, have previously been suggested as salivary biomarkers for pig welfare and our findings reinforce this.

### Downregulated proteins

Salivary proteins that were found in lower concentrations after chronic exposure to stress are chitinase, lipocalin-1, long palate lung and nasal epithelium protein 5, odorant-binding protein, vitelline membrane outer layer protein 1 homolog, and an uncharacterised protein that was a homolog of the vomeromodulin-like protein (*Ursus maritimus*). The latter was never identified in porcine saliva before.

#### PRM-validated downregulated proteins

In our study, chitinase was significantly decreased in the saliva of piglets exposed to stressors for three weeks. The family of chitinases are involved in inflammation, tissue remodelling and injury, and higher serum concentrations are associated to human diseases such as asthma (previously reviewed [91]). Importantly, the interpretation of these results must be performed with care. An effect of age on the concentration of this protein was observed in both treatment groups. However, this effect of age was lower in the stressed group leading to significantly lower levels of chitinase on day 28. Further investigation is needed to enable comparisons between different age groups.

Two members of the lipocalin family displayed a similar profile, i.e., lipocalin-1 and odorant-binding protein. The concentrations of these proteins rose with age under normal circumstances but not in a stressful situation. In consequence, significantly lower levels were observed in the saliva of 28-day-old stressed piglets when compared to control animals. Lipocalin-1 is mainly secreted by the porcine lachrymal glands and the lingual von Ebner’s glands [92]. In addition, it was also detected in gland-specific saliva and had similar concentrations in both mandibular/sublingual saliva and parotid saliva [24]. It protects the epithelia due to its role in the nonimmunological defence against micro-organisms and viruses and by controlling inflammatory processes [93]. Stress-related immunosuppression could explain these lower values [10, 11]. Of note is that the piglets in our study did not reach sexual maturity yet. However, very recently, it has been discovered that the concentration of lipocalin-1 in saliva fluctuates throughout the oestrus cycle, making interpretation of this biomarker difficult in sows [68]. Odorant-binding proteins are expressed by glands of the nasal cavity in vertebrates [94], but have also been found in gland-specific saliva of pigs, both in mandibular/sublingual saliva and in lower concentrations in parotid saliva [24]. These proteins are involved in mediating olfactory transduction, in chemical communication, and pre-mating recognition processes through pheromones [94, 95]. The suppression of this protein by a stressor could be linked to the negative effect of stress on reproduction capacity [14, 15]. Additionally, an increase in oxidative products associated with stress could contribute to its decrease [96]. The concentrations of both lipocalin-1 and odorant-binding protein were described to decrease after acute stress [46, 47], but also during disease [62]. The values of odorant-binding protein on day 28 correlated with all other PRM-validated proteins, except with alpha-2-HS-glycoprotein, and correlated with weight gain during the experiment (S1 Fig). This protein is locally expressed and secreted in the oral cavity and does not originate from the bloodstream. Its concentration can, therefore, not be altered by potential blood contamination of the sample. The only disadvantage of odorant-binding protein is that its concentration increases with age under non-stressed conditions, as those of lipocalin-1 and chitinase, making interpretation of results challenging. It is thus important to investigate the effect of age on these candidate biomarkers.

The vomeromodulin-like protein has similarities to odorant-binding protein, both in function and location [97, 98]. It is therefore, not surprising that the concentrations of this protein responded similarly to the exposure to stressors as the proteins of the lipocalin family. To our knowledge, no association between the vomeromodulin-like protein and stress has been described in studies using vertebrates.

Long palate lung and nasal epithelium protein 5 is the last protein with significantly lower values in the saliva of pigs reared under stressful conditions. Like the previously described lipocalin proteins, concentrations of this salivary protein rose with age under normal conditions, as in the control piglets, but failed to do so in response to the exposure to stressors. Knowledge about the function of this protein is scarce. However, like the parotid secretory protein, it is a member of the PLUNC family [81, 99]. Further insight into the function of this protein is advisable before this protein can be used as a biomarker for stressor exposure.

#### Non-validated upregulated proteins

Vitelline membrane outer layer protein 1 homolog was previously found in porcine saliva [24], but never concerning stress.

### General conclusion

Even though much insight into the salivary proteome of pigs and how these proteins respond to stressful conditions has been gained, our study has some drawbacks. In this study, we only investigated female animals. To verify whether our findings can be generalised to both sexes, our results should be validated in males, both chemically castrated and uncastrated. Additionally, as mentioned before, the effect of aging under normal conditions should be studied before different age groups can be compared correctly. It is important to know until which age the concentrations rise when they plateau, and whether the start of this plateau is similar for each candidate biomarker. These questions still need to be answered before the candidate biomarkers can be used in practice to identify welfare problems at the farm or facilitate research to optimise rearing conditions.

Nonetheless, not a single biomarker but rather a set of different proteins needs to be analysed to identify a complex problem such as chronic stress. On the one hand, it is known that different stressors elicit different responses in biomarkers (e.g. [28]). To reduce the chance of false negatives or false positive identifications, proteins linked to different pathways and processes should be analysed. However, also different individual coping mechanisms of the individual to the same stressor are reported (e.g. [100]). On the other hand, variation can arise from different sample processing methods, salivary flow rates or sample contamination (e.g. with blood, food or dirt).

Nevertheless, a possible set of biomarkers could include alpha-2-HS-glycoprotein, i.e., the confirmed upregulated protein whose abundance was affected even at the early timepoint, and odorant-binding protein, i.e., a downregulated protein that correlated to other physiological parameters. Chitinase and lipocalin-1, both linked to immunity, could be added to the set. Since chitinase has the least variation, it might be the preferred one out of the two. Lastly, long palate lung and nasal epithelium protein 5 is promising since this biomarker showed the highest difference between normal and stressful conditions and had the least variation. Additionally, to improve their use in stress evaluation, it is advisable that these proteins can be easily detected using antibody-based techniques.

To conclude, chronic exposure to different stressors altered the salivary proteome of piglets. Shotgun analysis using tandem mass spectrometry performed on saliva samples taken after three weeks of stress exposure identified 392 proteins, of which 20 proteins displayed significantly altered concentrations. Targeted PRM analysis confirmed that alpha-2-HS-glycoprotein was upregulated in the stressed group after one and three weeks, while odorant-binding protein, chitinase, long palate lung and nasal epithelium protein 5, lipocalin-1, and BLAST: vomeromodulin-like protein were present in lower concentrations in the saliva of the stressed pigs, however only after three weeks. The affected proteins could be used as salivary biomarkers after further validation to identify welfare problems at the farm and facilitate research to optimise rearing conditions.

## Supporting information

S1 Table Inclusion list. (XLSX)

S2 Table List of identified proteins. (XLSX)

S1 Fig Correlation matrix. (DOCX)

S1 Graphical abstract (TIF)
