# Supplementary material for: Chronic exposure to multiple stressors alters the salivary proteome of piglets
Source: PLoS One. 2023 May 26;18(5):e0286455. doi: 10.1371/journal.pone.0286455 (PMC10218721; doi:10.1371/journal.pone.0286455)

**Prims et al., 2023. Chronic exposure to multiple stressors alters the salivary proteome of piglets**

**Supporting information 3.** **Correlation matrix.** Correlation between the determined physiological parameters being the concentration of cortisol in hair and saliva and the weight gain during the three weeks of the experiment on the one hand, and the eight proteins validated by PRM, plus serotransferrin as an indicator for possible blood contamination, at the other hand side. A *P*-value smaller than 0.05 indicates a significant correlation between two parameters. The Spearman’s rho (ρ) specifies the strength of the relation which can be either positive or negative.


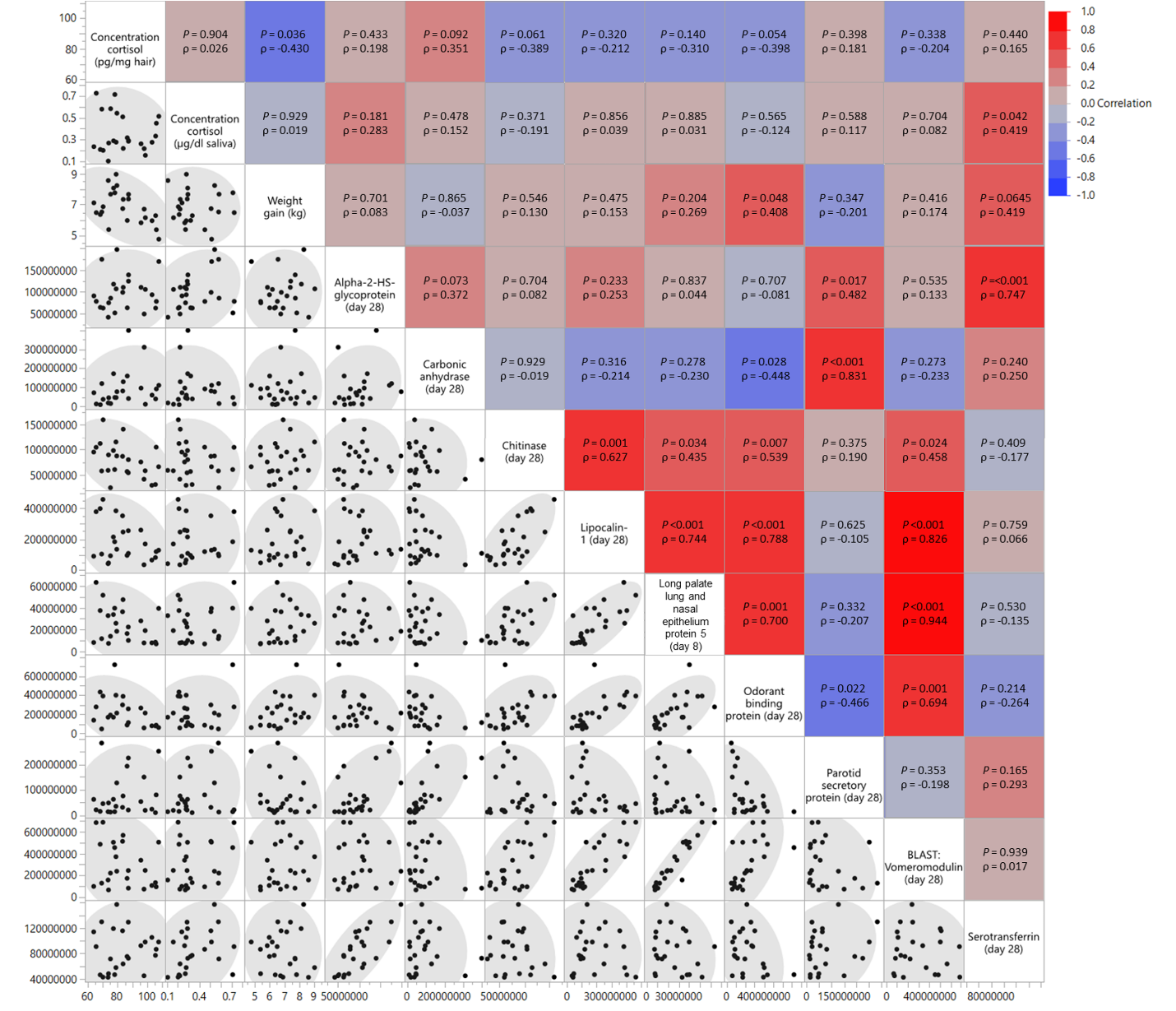

Supplement: S1 Fig — (DOCX) [file pone.0286455.s003.docx]
