# Supplementary figures and images for: Chronic exposure to multiple stressors alters the salivary proteome of piglets
Source: PLoS One. 2023 May 26;18(5):e0286455. doi: 10.1371/journal.pone.0286455 (PMC10218721; doi:10.1371/journal.pone.0286455)

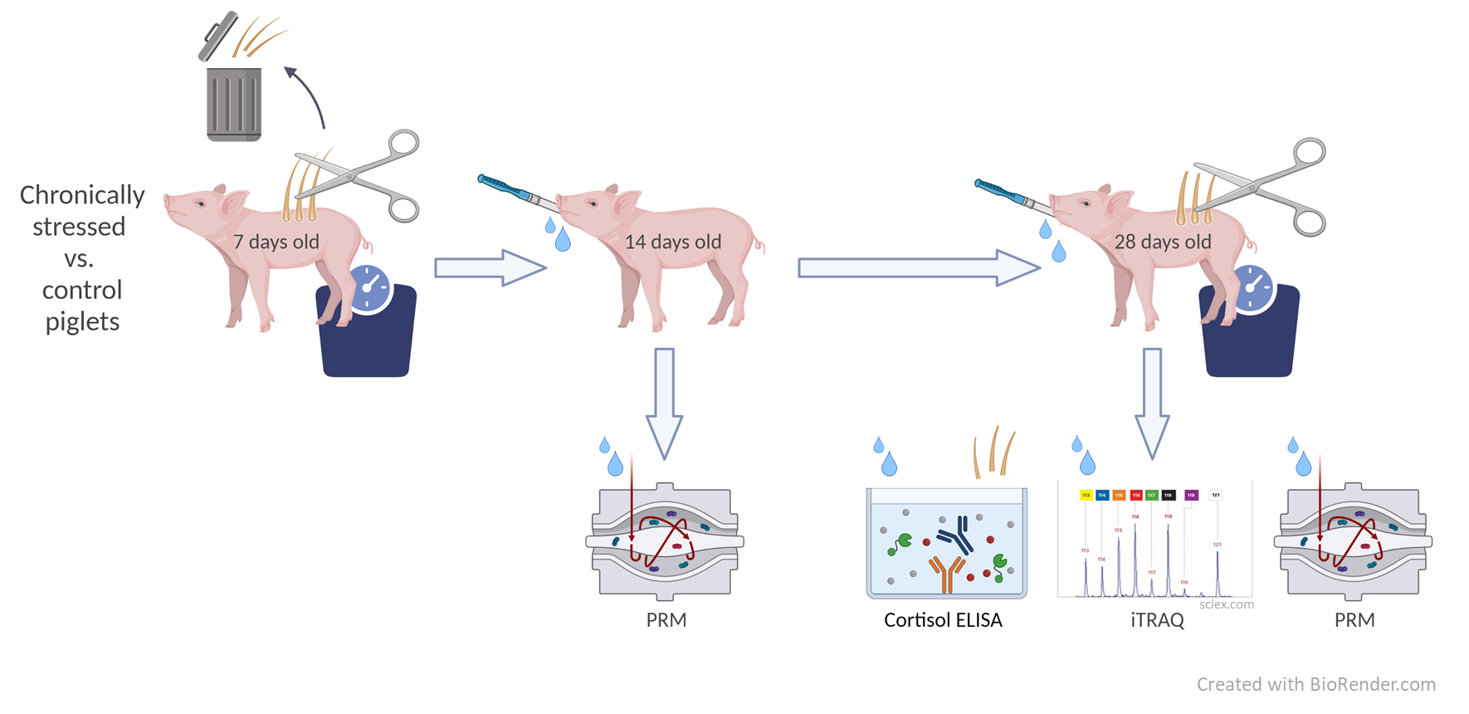

Supplement: S1 Graphical abstract — (TIF) [file pone.0286455.s004.tif]
